# Translational efficiency guides microbial community remodeling

**DOI:** 10.1080/19490976.2026.2736907

**Published:** 2026-09-19

**Authors:** Qi Xiang, Yanan Li, Jingpeng Yang

**Affiliations:** a State Key Laboratory of Microbial Technology, School of Food Science and Pharmaceutical Engineering, Nanjing Normal University, Nanjing, Jiangsu, People's Republic of China

**Keywords:** Microbial Interaction and Niche Determination (MIND), metagenomics, community remodeling, resource competition

## Abstract

While metagenomics provides compositional insights, its correlative nature limits causal community remodeling. Overcoming this, in a recent *Cell* study, Moyne et al. introduced the Microbial Interaction and Niche Determination (MIND) framework. By leveraging translational efficiency to map resource competition and niche partitioning, MIND establishes a mechanistic blueprint for rational engineering.

A fundamental bottleneck currently constrains microbiome science: the capacity to profile microbial communities has vastly outpaced the ability to mechanistically predict their dynamics.^1^ While metagenomics and metabolomics offer unprecedented resolution in mapping taxonomic composition and metabolic output, these approaches remain inherently descriptive and correlative. Consequently, although associations between microbial abundance and host or environmental variables are frequently observed, the underlying causal mechanisms remain largely elusive. This limitation is particularly evident in rational intervention design; for instance, while conventional omics data can identify metabolites associated with a beneficial commensal like *Faecalibacterium prausnitzii*, it cannot reliably predict which specific substrate addition would selectively enrich this taxon without inducing unintended, systemic perturbations across the broader community. The foundational rationale for designing synthesized communities relies heavily on integrating microbial genomic data with culturomics-based validation and *in vivo* animal models. However, there is a pronounced tendency to emphasize demonstrable phenotypic outcomes at the expense of theoretical robustness, specifically, whether the community composition is ecologically sound or if interspecies interactions align with the dynamics of the human gastrointestinal tract. This approach inherently predisposes artificially synthesized communities to manifest discrepant efficacies across *in vitro* and *in vivo* contexts.^2^ ^,^ ^3^

This disconnect between correlation and causation stems largely from the absence of direct functional metrics capable of quantifying a microorganism’s real-time metabolic demands within a community context. While genomics delineates functional potential and transcriptomics reflects transcriptional preparedness, neither equates to a cell’s active functional prioritization. Because protein translation is the most energetically demanding cellular process, bacteria must strictly allocate finite ribosomal resources to prioritize the synthesis of proteins essential for immediate survival and competition. This critical dimension of translational regulation has long remained a blind spot in community-level measurements, a gap only recently bridged by the advent of metaRibo-seq technology.^4^

In a recent study published in Cell,^5^ Moyne and colleagues elegantly bridge this gap by introducing the MIND (Microbial Interaction and Niche Determination) framework (Figure 1). This approach is predicated on two foundational premises. First, microorganisms exhibiting similar translational efficiency profiles constitute competitive functional guilds, as they allocate finite ribosomal resources toward overlapping metabolic pathways. Second, the translational prioritization of specific substrate transporters serves as a direct, quantitative readout of a microbe’s actual substrate preference within a complex community, essentially defining its ecological niche. By integrating interaction inference with niche determination, MIND not only maps competitive networks but also provides an actionable framework for rationally designing targeted interventions, such as the precise addition of prebiotics to selectively modulate community structure.

**Figure 1.**
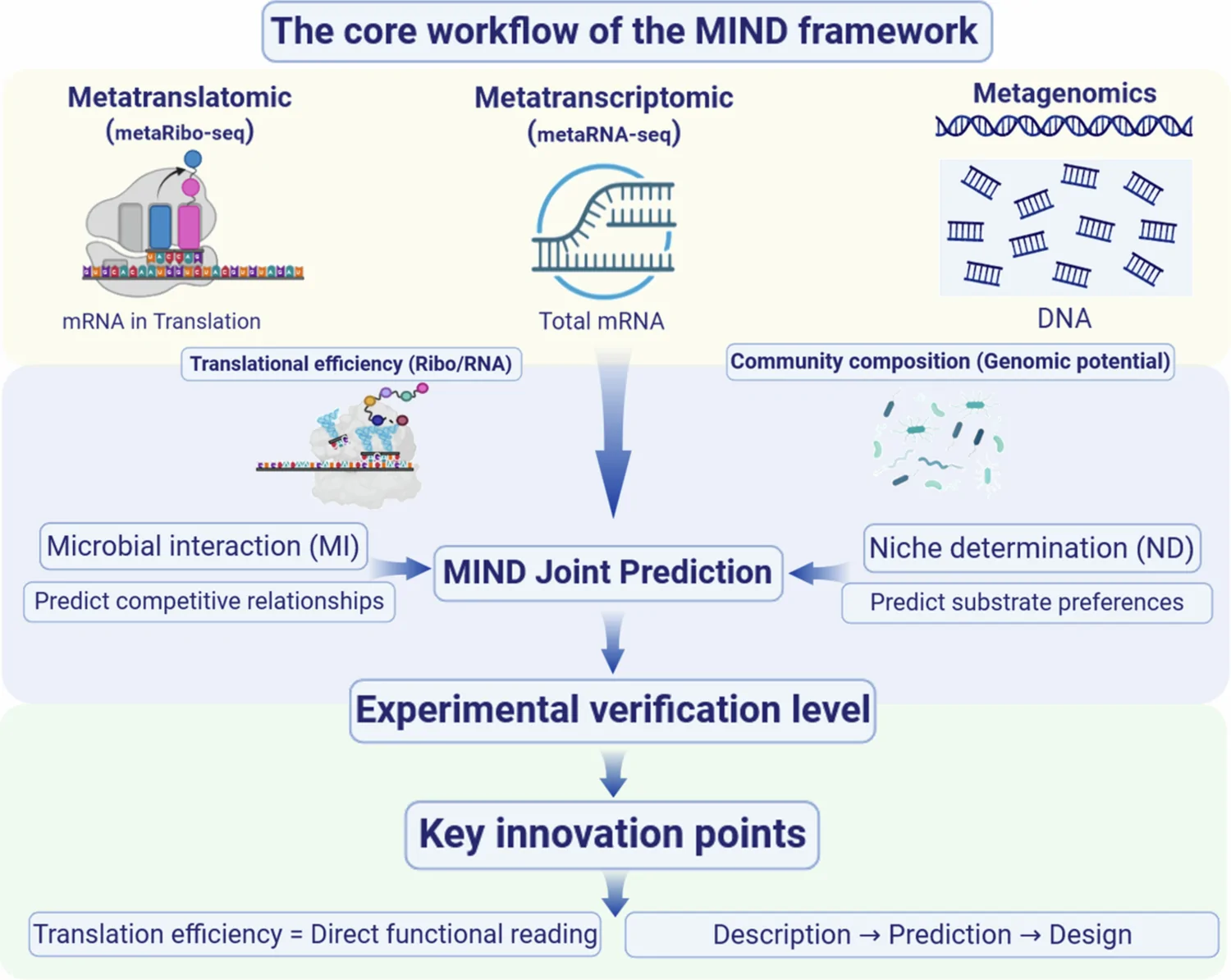
MIND Framework: From Translation Efficiency to Precise Microbiome Regulation.

A notable strength of this study lies in its rigorous, multi-tiered validation strategy. The core principles of MIND were first benchmarked in a defined synthetic community (SynCom) comprising 16 rhizosphere isolates. By aggregating translational efficiency (TE) across 275 KEGG metabolic pathways, the researchers constructed distinct functional profiles for each member. The competitive interactions predicted from TE profile similarity were robustly corroborated through systematic dropout experiments: the removal of a specific strain consistently resulted in the significant expansion of its nearest neighbor within the computed functional space. Notably, this TE-based predictive accuracy far surpassed that of models relying solely on metagenomic or metatranscriptomic data, conclusively demonstrating the necessity of capturing translational regulation to infer mechanistic ecological interactions.

Crucially, the niche determination (ND) component of MIND translates functional predictions into actionable interventions. As the study emphasizes, although bacteria may possess broad metabolic capabilities in axenic culture, they strictly constrain their translational investment to a narrow subset of transporters within a community context. This rigorously defined allocation constitutes their realized ecological niche. Consequently, the targeted supplementation of specific substrates (e.g., ribose or glutathione) selectively enriches primary targets exhibiting high translational efficiency for the corresponding importers. Concurrently, secondary targets, competitors occupying the same functional guild, are suppressed via competitive exclusion. This dual-action mechanism, simultaneously achieving targeted enrichment and competitor suppression, establishes a robust paradigm for rational, ecologically grounded community remodeling.

The broader significance of the MIND framework lies in its robust scalability, demonstrating that ecological principles derived from simplified synthetic consortia can be reliably extrapolated to complex, real-world ecosystems. Across highly diverse soil microcosms, human fecal assemblages, and in vivo host-associated environments, MIND consistently predicted community responses to targeted prebiotic interventions. A particularly compelling proof-of-concept is the in vivo targeting of *Faecalibaculum rodentium* in a murine model. Guided by MIND’s niche predictions, researchers selectively enriched this poorly characterized, hard-to-culture commensal via lactose supplementation, circumventing the need for prior metabolic knowledge. This approach fundamentally shifts the paradigm for microbiome engineering: the manipulation of recalcitrant microorganisms can now be driven by *in situ* functional profiling rather than empirical trial-and-error methodologies.

Despite its significant predictive capabilities, the current boundaries of the MIND framework delineate clear trajectories for future research. First, by predominantly capturing competitive interactions, a highly actionable lever for intervention, MIND currently lacks the mechanistic resolution to elucidate positive interactions, such as mutualism and cross-feeding, which are equally vital to overall community stability. Second, the predictive fidelity of the framework is inherently constrained by the quality of transporter annotations; consequently, its application to diverse environmental microbiomes is currently bottlenecked by the sparse functional characterization of uncultured taxa. Finally, when competing taxa exhibit overlapping substrate preferences, MIND cannot definitively resolve the ultimate competitive winner. As suggested by the authors, integrating MIND with genome-scale metabolic models (GEMs) represents a highly promising strategy to disentangle such predictive ambiguities. Importantly, to ensure this tool is accessible to the broader research community, the authors have made the original code publicly available. Original code has been deposited at https://github.com/ZenglerLab/MIND-and is publicly available as of the date of publication.

Notwithstanding these limitations, the MIND framework constitutes a paradigm shift in microbiome science. It facilitates a critical transition from correlational descriptions to causal predictions, and from passive observation to rational design. By coupling translational efficiency, a proxy intimately linked to cellular physiological imperatives, with ecological interaction networks, this study elevates precision microbiome engineering from a theoretical concept to a highly actionable methodology. Looking ahead, integrating multidimensional interaction signals, such as cross-feeding metrics, alongside increasingly comprehensive genomic annotations, could solidify MIND as a foundational platform for the rational manipulation of microbiomes across both clinical and environmental domains.

## Data Availability

Not applicable.
